# Oncogenic Fusions in NSCLC: From Mechanisms to Clinical Applications

**DOI:** 10.3390/ijms26083802

**Published:** 2025-04-17

**Authors:** Nyein Wint Yee Theik, Suset Almuinas De Armas, Daniel Rosas, Amy Kiamos, Nyein Nyein Thaw Dar, Ahmed Shoreibah, Atif Hussein, Luis E. Raez

**Affiliations:** 1 Memorial Healthcare System, Internal Medicine Residency Program, Pembroke Pines, FL 33028, USA; salmuinasdearmas@mhs.net (S.A.D.A.); ashoreibah@mhs.net (A.S.); 2 Memorial Cancer Institute, Memorial Healthcare System, Hematology-Oncology Fellowship Program, Pembroke Pines, FL 33028, USA; akiamos@mhs.net (A.K.); nthawdar@mhs.net (N.N.T.D.); 3 Memorial Cancer Institute, Memorial Healthcare System, Florida Atlantic University (FAU), Hollywood, FL 33021, USA; ahussein@mhs.net; 4 Thoracic Oncology Program, Memorial Cancer Institute (MCI), Florida Atlantic University (FAU), Hollywood, FL 33021, USA; lraez@mhs.net

**Keywords:** NSCLC, oncogenic driver, mutation, ALK, ROS1, RET, NTRK, TKI

## Abstract

Non-small cell lung cancer (NSCLC) is operated commonly by diverse genetic alterations, and oncogenic fusions represent a significant therapeutic role. Common fusions include ALK, ROS1, RET, and NTRK, signaling pathways in tumorigenesis. Recent advances in investigating tumor molecular biology include underlying fusions, including chromosomal rearrangements, highlighting their role as oncogenic drivers. The development of targeted therapies, such as tyrosine kinase inhibitors (TKIs), has impacted most patients’ NSCLC treatment. Despite the greater profiles, such as remarkable efficiency and tolerable side effects compared to traditional chemotherapy, challenges, such as acquired mutations, lead to more ongoing research-optimized future NSCLC therapies.

## 1. Introduction

Worldwide, NSCLC ranks among the primary reasons for cancer deaths, yet it presents a major treatment complexity. Before recent breakthroughs in medical knowledge, the primary treatment approach remained cytotoxic chemotherapy. However, it managed to extend patient survival times using multiple therapeutic agents, yet these medications brought intense side effects and resistance development. NSCLC treatment has evolved through molecular biology advancements to provide scientists insight into the genetic origins of NSCLC.

Researchers have identified oncogenic fusions among genetic rearrangements that drive cancer development as one of their most important discoveries. These functions are carried out by genes associated with c-ROS oncogene 1 (ROS1), neuregulin-1 (NRG1), anaplastic lymphoma kinase (ALK), neurotrophic tyrosine receptor kinase (NTRK), and epidermal growth factor receptor (EGFR) [1]. These fusions assist both in cancer cell formation and aid physicians with treatment decision making while serving as predictive indicators for therapy results. Advanced diagnostic methods such as immunohistochemistry (IHC), fluorescence in situ hybridization (FISH), and next-generation sequencing (NGS) enable high levels of fusion detection [2].

The review proceeds to examine how regular oncogenic fusion events maintain tumor progression throughout tumorigenesis. The review also examines how molecular discoveries have led to existing targeted therapeutic approaches as well as upcoming treatment methods. Our analysis establishes that oncogenic fusion studies create a connection between laboratory findings and medical treatments, which produces significant improvements in NSCLC care.

## 2. ALK Rearrangements

The occurrence of ALK rearrangement in NSCLC results when the ALK gene combines with EML4 or another gene due to chromosomal inversion on chromosome 2. The combination of these genes produces an active fusion protein named EML4-ALK that drives cancer cell multiplication. A chromosomal inversion into the short arm of chromosome 2 creates tumor development drivers when it places the EML4 gene adjacent to the ALK gene’s kinase region [3].

Research shows that ALK tyrosine kinase rearrangements exist in 4 percent of U.S. NSCLC adenocarcinomas, while they occur most frequently in non-smokers and younger individuals [4]. According to the crizotinib patient database, adenocarcinoma made up 97 percent of 255 cases with an ALK fusion oncogene presence [5]. The age distribution of participants within this research database included people from 21 to 82 years old, and their median age was 52 years [5]. Research findings indicate that ALK rearrangement exists at low rates in cases of squamous cell carcinoma (SCC) [6]. Medical tests to determine ALK gene rearrangements represent standard care practice for all patients with late-stage or metastatic non-squamous NSCLC at any stage of their smoking history [7].

The detection of ALK translocations requires any combination of FISH, IHC, or NGS panels [8]. The FDA-approved immunohistochemical test serves as a viable approach for ALK gene mutation screening to diagnose patients lacking NGS testing options within low- and middle-income medical settings [9]. When combined with FISH tests and IHC screenings, it has been shown that advanced-stage NSCLC patients who demonstrate ALK gene rearrangements will typically respond well to ALK tyrosine kinase inhibitor therapy.

### ALK Inhibitors

Approval granted by the FDA indicates that alectinib functions as the initial ALK-positive metastatic NSCLC agent while being effective for crizotinib progression cases [10]. Clinical trial results demonstrate that alectinib treatment for NSCLC patients delivered an abbreviated risk to disease worsening or mortality by 53 percent (HR 0.47, 95% CI 0.34–0.65) with a median progression-free survival (PFS) of 18 months versus 11.1 months in crizotinib patients [11]. Alectinib extended the CNS progression time for the general population by a percentage of 0.16 (95% CI 0.10–0.28) [11]. Alectinib outperformed crizotinib regarding patient outcome since subjects treated with alectinib survived progression free for 35 months while crizotinib subjects survived for 11 months (HR 0.43) [12]. During the phase III ALESIA study, researchers evaluated alectinib as a treatment for crizotinib in Eastern Asian patients with untreated ALK-positive NSCLC and determined that alectinib reduced the risk for progression or death (HR 0.22, 95% CI 0.13–0.38) [13]. The most common side effects of alectinib treatment when compared to crizotinib therapy involve anemia with myalgia and elevated bilirubin along with weight gain and phototoxicity [13].

Brigatinib represents a first-line option of treatment for ALK-positive, advanced NSCLC patients because it attacks many ALK mutations better than crizotinib [14]. The FDA approved brigatinib for treating stage IV NSCLC based on data from the ALTA 1L trial, which established its similar effectiveness to alectinib during initial treatment of NSCLC. The phase III randomized trial, including 275 patients with advanced treatment-naïve ALK-positive NSCLC, revealed that brigatinib produced better PFS outcomes than crizotinib (12-month PFS rate: 67 percent versus 43 percent; HR 0.49, 95% CI 0.33–0.74) after a median follow-up of 9–11 months [14]. The pulmonary toxicity risk associated with brigatinib treatment affects only a limited number of patients, yet step-up dosing strategies starting at 90 mg once daily for seven days followed by a dose increase to 180 mg once daily reduce this risk [15]. Brigatinib generates increased creatinine kinase levels in addition to causing coughing along with hypertension rise and interstitial lung disease or pneumonitis manifestations according to [15].

The use of lorlatinib stands as an initial treatment option specifically for patients with ALK-positive advanced or metastatic NSCLC. Research published at ASCO 2024 together with the World Lung Conference (phase III CROWN trial) demonstrated that treatment-naïve patients with ALK-positive stage IIIB/IV NSCLC received either crizotinib or lorlatinib out of 296 participants [16,17]. The results after an 18-month follow up showed that lorlatinib provided no evaluable median PFS versus the 9.3 months achieved by crizotinib (HR 0.28, 95% CI 0.19–0.4), while the five-year PFS rate reached 60 percent for lorlatinib and only 8 percent for crizotinib [16,17]. Treatment with lorlatinib produces hypercholesterolemia and hypertriglyceridemia alongside neurocognitive side effects relative to crizotinib administration. Research findings indicate that lorlatinib gained medical approval as initial therapy for ALK-positive NSCLC because it exhibits a five-year PFS rate [17].

Clinical trials showed ceritinib works as the second-generation ALK TKI and remains FDA approved for the treatment of metastatic ALK-positive NSCLC patients while displaying potency levels about 20 times stronger than crizotinib. Scientists conducted ASCEND-4 research with 376 untreated patients who possessed NSCLC with ALK-positive status receiving ceritinib therapy or opposed to pemetrexed along with a platinum-based drug [18]. Research shows that ceritinib outperformed standard chemotherapy treatment for PFS (16.6 months compared to 8.1 months; HR 0.55, 95% CI 0.42–0.73) while providing a better ORR at 72.5 percent versus 26.7 percent with duration of response at 23.9 months versus 11.1 months [18]. The initial trials using a 750 mg daily ceritinib dose administered under fasting conditions generated GI toxicity, which required an open-label randomized trial with a 450 mg daily dosage taken with food to reduce GI adverse effects [19].

The multitargeted TKI agent crizotinib brought substantial benefits for patients with advanced NSCLC who are ALK positive through its role as the first ALK inhibitor [20]. The randomized trial administered crizotinib to ALK-positive chemotherapy-naïve NSCLC patients, leading to extended PFS results (median 10.9 months versus 7 months; HR 0.45, 95% CI 0.35–0.60, ORR 74 percent versus 45 percent) and prolonged overall response rates at a median follow-up duration of 17 months when compared to chemotherapy-based therapy [20,21]; however, the availability of other mentioned TKIs renders crizotinib less desirable for first-line use because of its inferior PFS and CNS penetration.

Ensartinib demonstrated better results than crizotinib as the first-line agent for advanced treatment-naïve ALK-positive NSCLC patients. A phase 2 trial known as eX-alt3 enrolled 290 patients with ALK-positive NSCLC patients to compare ensartinib treatment against crizotinib through risk reduction analysis that found PFS improving from 13 to 26 months (HR 0.51, 95% CI 0.35–0.72) [22]. Treatment with ensartinib causes both rash and transaminitis as common side effects [22].

## 3. NTRK Fusions

In cancer, NTRK gene fusions arise through chromosomal rearrangements, fusing the 3′ end of an NRTK gene (encoding the tyrosine kinase domain) with the 5′ end of a partner gene [23]. Research has extensively studied the ETV6-NTRK3 fusion that was originally detected in congenital fibrosarcoma because other partnerships, like TPM3-NTRK1 and TFG-NTRK1, exist in both lung cancer and multiple solid tumor types [24]. The occurrence of NTRK fusions presents itself as a very uncommon event in lung cancers, affecting between 0.2 and 3% of NSCLC patients. Patients who lack EGFR, ALK ROS1, and KRAS driver mutations tend to have these fusions along with younger age groups, non-smokers, and those with adenocarcinoma pathologies [25].

NGS along with FISH enables scientists to detect rare fusions that exist within lung cancers. Lung cancer exhibits low NTRK fusion frequency but higher significance because these fusions possess a high dependency on oncogenesis. Preclinical research shows NTRK fusion-positive lung cancer cells strongly depend on TRK fusion protein function for cell growth and survival, which renders them sensitive to TRK inhibitor treatment [26].

Detection methods of NTRK fusions require accurate results to direct the appropriate use of TRK inhibitors. This set of diagnostic methods includes IHC as a first screening approach to detect TRK protein overexpression but demonstrates insufficient specificity. FISH enables researchers to identify gene rearrangements, yet its ability to detect specific fusions depends on the designed probes. The evaluation method Reverse-Transcriptase Polymerase Chain Reaction (RT-PCR) detects unique fusion transcripts, yet it needs knowledge about involved fusion partners beforehand. Next-generation sequencing stands as the most wide-ranging technology to identify NTRK fusions because it detects known and novel fusions within a single testing platform.

### TRK Inhibitors

The medical care for cancers containing NTRK fusions saw substantial improvement through the development of TRK inhibitors. Larotrectinib together with entrectinib represents the first group of TRK inhibitors, which demonstrate outstanding treatment results for NTRK fusion-positive tumor patients, including patients with lung cancer [27]. Larotrectinib showed excellent results as a treatment for NTRK fusion-positive cancers and specifically came recommended for NSCLC patients during clinical trials. A major aspect of two global multicenter clinical trials involved an ORR evaluation of 15 patients for which the response rate reached 73% (95% CI, 45 to 92). The clinical outcomes of interest showed a median response duration of 33.9 months (95% CI 5.6 to 33.9) and a median PFS duration of 35.4 months (95% CI 5.3 to 35.4). Moreover, the median overall survival reached 40.7 months (95% CI 17.2 to not estimable). The response rate for patients who had CNS metastases at baseline measurement was 63% for treatment. Patients experienced mostly first-degree and second-degree adverse reactions according to the authors [28]. The most recent analysis of the clinical trial from May 2024 showed that DOR, PFS, and OS rates at 48 months reached 47% (95% CI 19–76), 34% (95% CI 11–56), and 38% (95% CI 13–64), respectively [29].

Metabolic research shows entrectinib succeeds against NTRK fusion-positive cancers by achieving an approximately 57% overall response rate within NSCLC. The molecule shows effective passage through the blood–brain barrier, so doctors can benefit from its application during CNS metastasis treatment within lung cancer patients [30]. The effectiveness of TRK inhibitors might lead to acquired resistance forming as a treatment limitation. Secondary mutations affecting the NTRK kinase domain prevent inhibitor binding by introducing G595R and G667C mutations within the domain.

Research on second-generation TRK inhibitors selitrectinib (LOXO-195) and repotrectinib (TPX-0005) has shown promising results in clinical trials as they bypass resistance mutations [31]. The TRIDENT trial enrolled *n* = 40 patients with locally advanced or metastatic solid tumors carrying NTRK fusions to investigate their response to these inhibitors [32]. The ORR for patients who received no prior treatment with TRK TKI reached 58% (95% CI, 41–73) following 17.8 months of the observation period. The cORR in the pretreated TRK TKI cohort reached 50% (95% CI, 35–65) during 20.1 months of median follow up. The treatment duration reached approximately twelve months, while patients experienced 9–11 months of disease progression-free period. The most frequently reported side effects were fatigue, dizziness, and nausea, which mostly emerged as grade 1 or 2 in severity.

Clinical trial TRIDENT demonstrated that repotrectinib provides an effective treatment choice for NTRK fusion-positive tumor patients along with satisfactory safety measures, thus offering valuable treatment options to this difficult patient group. The low prevalence of NTRK fusions shows the need for extensive genomic testing because targeted testing could overlook these vital although uncommon genomic alterations. The therapeutic breakthrough using TRK inhibitors generated enormous progress in NTRK fusion-positive cancer treatment, but related hurdles persist.

Research is underway to study the potential benefit of combining targeted and immunotherapy methods for NTRK fusion-positive cancers regardless of their TMB and PD-L1 expression levels due to immune checkpoint inhibitors’ success in NSCLC [33]. NSCLC patients with NTRK fusions make up a rare subgroup whose treatment response stands to benefit greatly from directed medications. Treatment of patients with NTRK gene fusions has completely changed due to the introduction of larotrectinib and entrectinib TRK inhibitors, which bring about long-lasting responses together with potential survival benefits. The scarcity of NTRK fusions as well as the eventual occurrence of resistance require ongoing investigations to develop better diagnostic procedures and innovative drugs and combination treatment approaches.

## 4. NRG1 Fusion

The discovery of NRG proteins occurred in 1992, and they execute dual functions by dictating early developmental cell differentiation while facilitating cellular connections between neurons, muscle cells, and mammary cells [34]. The ligand protein NRG1 belongs to the epidermal growth factor (EGF) protein family while existing as a product of the 8p12 chromosome NRG1 gene [35]. The N-terminal area of NRG1 generates different isoforms, which result in protein variation. The receptors of the ErbB signaling family, including EGFR and ErbB2 (HER2), as well as ErbB3 (HER3) and ErbB4 (HER4), have EGF-like structures, which enable them to bind NRG1 ligands [36]. The activation of PI3K/serine–threonine protein kinases (PI3K/AKT) together with MAPK pathways following ligand binding results in cell proliferation and promotes cellular migration and differentiation as well as survival [37].

Research on NRG1 fusion and its signaling mechanism in lung cancer tissues emerged during 2014–2015 alongside studies of adenocarcinoma transcriptome, which demonstrated CD74-NRG1 gene fusion [38,39]. The analysis performed by Gupta et al. detected NRG1 fusion occurrence in 0.15% of tumor specimens, and each single tumor type showed a prevalence rate of 0.30% for breast cancer and 0.26% for cholangiocarcinoma along with 0.23% NSCLC, 0.22% carcinoma of unknown primary, 0.19% pancreatic cancer, 0.17% ovarian cancer, 0.15% bladder cancer, 0.15% esophageal cancer, and 0.15% vulvar cancer [40]. The fusion events in the research involved 153 distinct partner proteins, while CD74 (12.37%) and SLC3A2 (8.13%) led the list followed by ATP1B1 (4.59%) and RBPMS (4.24%) [40]. Fusion partners additionally include SDC4, FGFR1, CADM1, DIP2B, F11R, FLYWCH1, ITGB1, KRAS, MDK, MRPL13, PLCG2, TNC, VAMP2, and VAPB [41].

NRG1 fusion occurs mostly in invasive mucinous adenocarcinoma (IMA) and acinar adenocarcinoma subtypes along with non-smoker female lung cancer patients [41,42]. Only a review from 2018 proposed the idea of NRG1 fusion being the opposite of KRAS mutations [43], although new research has shown their simultaneous presence. KRAS mutation triggers the cleavage of NRG1 from SLC3A2-NRG1 fusion while enhancing Ras/Raf/MEK/ERK and ErbB/PI3K/Akt/mTOR signaling to produce cell growth and proliferation [44]. Research studies indicate that NRG1 fusion exists independently of both EGFR mutation and ALK rearrangement in IMA patients [45]. Research has shown that a single case exists where patients demonstrated RALGAPA1-NRG1 fusion alongside ALK rearrangement as a resistance mechanism for ALK-targeted therapy, such as crizotinib, yet responded well to the treatment with afatinib [46]. Table 1 displays all documented NRG1 fusion partners that occur in lung cancer cases.

The detection of NRG fusion requires RNA sequencing instead of DNA sequencing because this fusion contains multiple gene isomers and extremely large intronic regions [47]. The clinical performance of targeted RNA sequencing surpasses that of FISH and IHC testing [48]. RNA sequencing identifies NRG1 fusions with greater effectiveness than DNA sequencing approaches. The identification of new targetable gene fusions in NSCLC occurs through RNA sequencing when NGS analysis shows no driver oncogene [49].

### NRG1 Inhibitors

The NRG1 fusion type of lung cancer displays worse outcomes due to its resemblance to cancer stem cells and its resistance against both chemoimmunotherapy strategies [35,50,51,52]. The survival duration of patients carrying NRG1 fusion IMA was 51.9 months, while the survival data for patients without NRG1 fusion IMA remained un-reached according to Shin et al. [53]. This research showed identical recurrence-free survival results between mucinous adenocarcinoma patients and patients with non-mucinous adenocarcinoma [54]. Research findings from a multicenter retrospective study indicated that chemotherapy with platinum doublet produced a 13% ORR, while taxane-based follow-up platinum doublet obtained a 14% ORR, with chemoimmunotherapy and single-agent immunotherapy yielding 0% and 20% responses, resulting in a PFS of 5.8 months, 4.0 months, 3.3 months, and 3.6 months, respectively. The expression of PD-L1 remained low at 28%, and TMB measurement yielded a low median value of 0.9 mutations per megabase. Moreover, afatinib demonstrated an ORR of 25% together with a median PFS duration of 2.8 months [43]. The mix of existing mutations together with the specific partner fusion genes might influence disease prognosis while impacting chemoimmunotherapy responses, and researchers need to conduct further investigations in this field.

The therapeutic targets involving NRG1 ligand protein function for ErbB receptors include TKIs with ErbB receptor antibodies alongside inhibition techniques for the ErbB downstream pathway. Research data demonstrate the usefulness of GSK2849330 anti-Her3 antibody [55] and seribantumab along with zenocutuzumab (MCLA-128) bispecific humanized monoclonal antibody [56,57] and afatinib and tarloxotinib (pan-ErbB kinase inhibitor) [55,58]. The information regarding the clinical outcomes of targeted therapy appears in Table 1. The available data primarily consist of case reports and case series. The TKI drug afatinib irreversibly blocks the activity of ErbB receptors. Twelve patients treated with afatinib showed a median PFS outcome in NRG1 fusion NSCLC, which measured 3.5 months with a range from 0.6 to 16.5 months according to Duruisseaux et al. [59]. The treatment response durations of afatinib according to case reports typically extended between 6.5 and 12 months [60,61,62].

Tarloxotinib is a pan-ErbB kinase inhibitor, and currently, the RAIN (NCT03805841) trial is recruiting for a phase 2, open-label, single-treatment arm in cohort C with NRG1 and HER fusions, but the final analysis of safety and efficacy of cohort C is pending. Anti-Her3 antibody (GSK2849330) showed a more prolonged duration of response of up to 19 months [63], but no further clinical trials proceeded after the phase I trial. Another anti-Her3 antibody is lumretuzumab, and the combination of lumretuzumab and erlotinib in SLC3A2-NRG1 fusion disease maintains the stable disease for 4 months in case reports [64]. Patritumab (U3–1287; AMG-888) is also an anti-Her3 inhibitor, and the HERALD (NCT02134015) trial showed that the clinical benefit of patritumab plus erlotinib was found to have in high HER3 ligand heregulin (HRG) mRNA levels, which would be a predictive biomarker in future [65].

Seribantumab (MM-121) was granted by the FDA as fast track designation for NRG1-positive, locally advanced, or metastatic NSCLC after failure of immunotherapy based on the SHERLOC trial (NCT02387216). A recent case series study from the SHERLOC trial showed a deep and prolonged duration of response in NRG1 fusion NCSLC. For example, an SLC3A2-NRG1 fusion achieved a partial response with more than 35 months of duration of response [66]. CRESTONE trial (NCT04383210) interim analysis in 2022 showed a 90% disease control rate of seribantumab (CR = 1, PR = 2, SD = 6, PD = 1) in ten NSCLC patients with five different NRG1 fusion partners (ATP1B1, CD74, ITGB1, SDC4, SLC3A2). The ongoing duration of response is 6–8.5 months with well-tolerated adverse effects [67]. However, the trial was terminated in early 2024, and the final analysis has not been published yet.

Zenocutuzumab (MCLA-128) is a bispecific humanized monoclonal antibody and inhibits the heterodimerization of Her2 and Her3. In 2024, it was approved by the FDA for metastatic NRG1 fusion-positive solid tumors, especially for pancreatic and lung cancer after failure of standard of care. A case of CD74-NRG1 fusion NSCLC showed a partial response with an ongoing durable response >2 months in a basket phase 2 trial [57]. Currently, the phase I/II, open-label, multicenter (NCT02912949) trial is recruiting patients to evaluate the safety, efficacy, and pharmacokinetics of zenocutuzumab in NRG1 fusion harboring solid tumors. Interim analysis presented at the ASCO 2021 annual meeting by Dr. Schram showed that the ORR was 29% (seven out of twenty-four patients) in NSCLC. Other promising targeted therapies for NRG1 fusion are lapatinib, pyrotinib, trastuzumab, pertuzumab, and humanized Her3 monoclonal antibody (AV-203), a non-competing anti-HER3 antibody with high affinity to NRG1 ligand (9F7-F11). Most of the data are from preclinical studies, and the clinical efficacy data are not available yet.

**Table 1:** The NRG1 partner fusion sites reported in lung cancer. DR = duration of response, PR = partial response, PFS = progression-free survival, SD = stable disease.

Partner Gene	Chromosome Location	Function of Gene	Treatment	Treatment and Response	Reference
ATP1B1	1q24.2	Na^+^/K^+^ transport across membranes	Seribantumab	Disease control rate 90% DR ongoing 6–8.5 months [67]	[41]
CD74	5q33.1	MHCII chaperon Cell surface receptor	Afatinib	PR with DR 10 months [60] PR with DR 6.5 months [62] SD with DR 3 months [55]	[38,39]
			GSK2849330	PR with DR 19 months [55,63]	
			Zenocutuzumab (MCLA-128)	PR with DR >2 months (ongoing trial, NCT02912949) [57]	
			Seribantumab	Disease control rate 90% DR ongoing 6–8.5 months [67]	
			Afatinib+ Pyrotinib	PR with PFS 5 months [68]	
CADM1	11q23.3	Cell adhesion molecule 1			[43]
DIP2B	12q13.12	Embryogenesis organogenesis Lung maturation Axon growth			[43]
DPYSL2	8p21.2	Neuron growth			[41,43]
F11R	1q23.3	Epithelial tight junction Leukocyte migration Platelet receptor			[43]
FGFR1	8p11.23	Tyrosine kinase receptor			[43]
FLYWCH1	16p13.3	DNA repair			[43]
ITGB1	10p11.22	Cell adhesion Angiogenesis Neuro growth	Seribantumab	Disease control rate 90% DR ongoing 6–8.5 months [67]	[42]
KIF13B	8p12	Angiogenesis Intracellular trafficking			[69]
KRAS	12p12.1	Cell growth, maturation, and death			[43]
MDK	11p11.2	Cell growth, metastasis, and angiogenesis Inflammation Hippocampal development			[41]
MRPL13	8q24.12	Cell cycle progression Tumor cell proliferation Mitochondria integrity			[41,43]
NPTN	15q24.1	Cell-to-cell interaction Cell-to-substrate interaction	Afatinib (4th line)	PFS 14 months	[70]
PARP8	5q11.1	Post-translation protein modification			[41]
PLCG2	16q23.3	Immune system Intracellular signaling			[41]
RALGAPA1	14q13.2	Intracellular signaling			[46]
RBPMS	8p12	Cardiomyocyte contraction			[43,55]
ROCK1	18q11.1	Cell motility Autophagy Inflammation			[41]
SDC4	20q13.12	Cell signaling Cell proliferation, adhesion, and migration Angiogenesis	Afatinib	PR with DR 12 months [61]	[41,43]
			Seribantumab	Disease control rate 90% DR ongoing 6–8.5 months [67]	
SLC3A2	11q12.3	Transmembrane protein Highly expressed in cancer	Afatinib	PR with DR 12 months [60]	[41,43,53]
			Lumretuzumab + Erlotinib	SD with DR 4 months [64]	
			Seribantumabc	PR with DR >35 months [66] Disease control rate 90% DR ongoing 6–8.5 months [67]	
SMAD4	18q21.2	Transcription factor Tumor suppressor			[55]
THAP7	22q11.21	Negative regulation of histone acetylation			[55]
TNC	9q33.1	Tissue repair Wound healing			[41,43]
WRN	8p12	DNA repair, replication, and transcription Telomere maintenance			[71]
VAMP2	17p13.1	Epidermal differentiation Carcinogenesis			[54,72]

## 5. RET Translocation

In the 1980s, the Rearranged during Transfection (RET) gene was identified as a proto-oncogene [73]. The receptor tyrosine kinase (RTK), encoded by the RET gene on chromosome 10q11.2, is essential for the development of the genitourinary, enteric neurological, and neuroendocrine systems in the embryo [74]. Unlike other RTKs, RET can be activated without direct binding to its ligands, such as persephin, neurturin (NRTN), glial cell line-derived neurotrophic factor (GDNF), and artemin (ARTN) [75]. Instead, the ligands attach to GDNF family receptors, which causes RET to be recruited extracellularly. This, in turn, causes the intracellular tyrosine kinase domain to become autophosphorylated, which activates downstream signaling pathways such as MAPK, P13K, JAK-STAT, and PKA [75,76]. The downstream-activated signaling pathways support cell survival, growth, differentiation, and proliferation [75,76].

RET oncogenic activation is driven by two primary mechanisms: mutations or chromosomal rearrangements [75]. RET proto-oncogene mutations and fusions cause gain-of-function activity, leading to uncontrolled activation of the downstream signaling cascades irrespective of ligand binding [77]. The constantly activated pathways lead to unregulated cell proliferation, subsequently causing tumor genesis. RET variations have been discovered in several types of solid malignant tumors including breast, colon, renal, pancreas, thyroid, and NSCLC [78]. RET mutations (both somatic and germline) are well described in the pathogenesis of medullary thyroid cancer (MTC). RET germline mutations are associated with multiple endocrine neoplasia (MEN) 2A and 2B syndromes, which are known for the increased risk for MTC and neuroendocrine malignancies [75,79]. In comparison, sporadic MTC is associated with RET somatic mutations in 40–60% of cases [79].

The RET gene plays an important role in precision medicine for NSCLC. It is recommended that all patients with NSCLC undergo testing to identify oncogenic drivers and possible targets for treatment therapy [80]. RET chromosomal rearrangements are found in 1–2% of NSCLC [79,80]. The most commonly identified RET gene fusions include KIF5B-RET, CCDC6-RET, and NCOA4-RET [80]. The development of RET inhibitors has shown impressive outcomes and is now established as a standard-of-care treatment option for lung and thyroid cancer.

### RET Inhibitors

In 2020, the FDA authorized selpercatinib, the first RET TKI with high selectivity. Selpercatinib was given to patients with RET fusion-positive non-small cell lung cancer (NSCLC) in the LIBRETTO-001 phase I/II open-label trial (69 patients were treatment naïve, 247 had previously received platinum-based chemotherapy) [81]. Treatment-naïve patients had an ORR of 84%, while platinum-pretreated individuals had an ORR of 61%. Patients without platinum had a DOR of 20.2 months, while those who had received platinum had a DOR of 28.6 months. Platinum-pretreated patients had a median PFS of 24.9 months, while treatment-naïve patients had a median PFS of 20.2 months. Selpercatinib’s intracranial ORR was 85% in 106 patients with detectable brain metastases (both platinum pretreated and treatment naïve) with a PFS of 19.4 months [81]. The most frequent grade 3 adverse events were QT prolongation, diarrhea, transaminitis, and hypertension [81].

The LIBRETTO-431 phase III trial evaluated selpercatinib as a first-line treatment for advanced RET fusion-positive NSCLC in comparison to platinum-based chemotherapy with or without pembrolizumab [82]. Selpercatinib showed a longer PFS of 24.8 months as opposed to 11.2 months in the group that received only chemotherapy. An identical PFS of 11.2 months was seen when pembrolizumab was added to chemotherapy. The DOR for selpercatinib was higher at 24.2 months than it was for the chemotherapy group, which was at 11.5 months. The intracranial ORR for selpercatinib in patients with baseline brain metastases was 82.4% (with a complete response rate of 35.3%), while the intracranial ORR for the chemotherapy group was 58.3% (with a complete response rate of 16.7%). Significantly, selpercatinib-treated individuals reported a lower quality of life (23%) than those receiving chemotherapy (36%) [82].

The most frequent side effects were the same as those of LIBRETTO-001. For stage 4 RET fusion-positive NSCLC, LIBRETTO-431 offered compelling evidence in favor of selpercatinib as a first-line therapy option [82]. Impressive long-term patient-reported outcomes (PROs) for RET-altered NSCLC, MTC, non-MTC thyroid cancer, and tumor agnostic (TA) patients from the LIBRETTO-001 trial were reported in a post hoc retrospective study by Raez et al. [83]. A Quality-of-Life Questionnaire Core 30 (QLQ-C30) was filled out by patients at baseline, two years later, and three years later. With ongoing selpercatinib therapy, patients in all groups reported better or stable QLQ-C30 scores (75% NSCLC, 81% MTC, 75% thyroid cancer, 40% TA). The median duration of improvement was between 1.9 and 28.2 months, while the median time to first improvement was between 2.0 and 19.4 months [83]. This study highlights important quality-of-life improvements with selpercatinib treatment.

In 2020, the FDA authorized pralsetinib, another highly selective RET TKI, for the treatment of NSCLC. Patients with RET fusion-positive NSCLC (29 treatment naïve, 92 with prior platinum-based chemotherapy) were given pralsetinib as part of the ARROW phase I/II open-label trial [84]. The ORR for patients who had never had treatment was 70%, with 11% exhibiting full response. The ORR was 61% in patients who had received platinum pretreatment, with 6% seeing a full response. Treatment-naïve individuals had a median PFS of 9.1 months, while platinum-pretreated patients had a median PFS of 17.1 months. Five–six percent of the nine patients with detectable brain metastases who received platinum pretreatment achieved an intracranial response, including three full responses [84]. Anemia, pneumonia, pneumonitis, hypertension, and neutropenia were the most frequent grade 3 adverse events [84].

Both selpercatinib and pralsetinib have shown promising outcomes in RET fusion-positive NSCLC, especially in the setting of CNS activity. Studies have also shown efficacy for both medications in RET-altered thyroid cancer [85,86]. Although RET TKI treatment responses have been favorable, the duration of the response can be halted by acquired resistance [87]. A few studies have shown that mutations in the RET kinase domain located in the solvent Gly-rich group (L730, E732, V738), gatekeeper residue (V804), and large C-terminal lobe cause interference, with drug binding ultimately leading to resistance [88]. Vepafestinib (TAS0953/HM06) is a next-generation RET inhibitor with a unique binding mechanism. It has strong selectivity for RET and is effective against common resistance mutations (RETL730, RETV804, and RETG810) [89]. Compared to existing RET inhibitors, vepafestinib also has better drug absorption in the brain. It is important to re-biopsy and repeat molecular testing in patients who experience progression on RET TKIs to identify mechanisms of resistance and develop new ways to overcome them. There are several ongoing clinical trials to expand treatment options with other RET TKIs, such as cabozantinib and vandetanib [88]. Hopefully, with future research and further identification of resistance mechanisms selective RET, TKIs will continue to improve outcomes in RET-altered cancers.

## 6. ROS1 Mutations

Within the diverse molecular landscape of NSCLC, receptor tyrosine kinase (RTK) fusions, such as ROS1 gene rearrangement, have gained significant attention due to their oncogenic role and implications for targeted therapies. ROS1 fusions are found in 1–2% of NSCLC cases, predominantly in non-smokers and younger patients with adenocarcinoma histology. The identification of ROS1 fusion proteins has led to the development of targeted therapies, significantly altering the treatment landscape for these patients.

A receptor tyrosine kinase associated with the insulin receptor family is encoded by the ROS1 gene. Constitutive activation of the kinase domain results from ROS1 fusions, which happen when the ROS1 gene on chromosome 6q22 rearranges with different partner genes, including CD74, SLC34A2, and TPM3. Through downstream pathways, like the PI3K/AKT and MAPK pathways, this ongoing signaling encourages cell division, survival, and metastasis [90]. TKIs can target ROS1 fusions, which are comparable to ALK rearrangements, another oncogenic driver in NSCLC. Even though ROS1 fusions are uncommon in NSCLC, identifying them is essential for individualized treatment strategies since individuals with these fusions benefit greatly from targeted medicines. The identification of ROS1 fusions in NSCLC relies on sensitive and specific diagnostic tools, including FISH, RT-PCR, and NGS [91]. FISH has traditionally been the gold standard for detecting ROS1 rearrangements; however, NGS platforms have gained widespread use due to their ability to simultaneously assess multiple genetic alterations, offering a more comprehensive molecular profile.

In recent years, liquid biopsy approaches, such as circulating tumor DNA (ctDNA) analysis, have emerged as a non-invasive tool for detecting ROS1 fusions, particularly in patients where tissue samples are limited [92]. However, liquid biopsies are currently limited to ctDNA and do not capture RNA, making it challenging to detect certain fusions like ROS1. This highlights the continued importance of NGS RNA analysis in tissue samples, which remains a more reliable method for identifying fusions. Liquid biopsies offer valuable insights for real-time monitoring of treatment response and detection of resistance mutations, aiding in clinical decision making.

### ROS1 Inhibitors

ROS1-positive NSCLC treatment has been transformed by TKIs that target ROS1 rearrangements. The first medication approved by the FDA for ROS1-positive NSCLC was crizotinib, a multitargeted TKI that was first created for ALK-positive NSCLC. This approval was based on the PROFILE 1001 study. In this study, crizotinib showed a median PFS of 19.3 months and an ORR of 72% [93]. However, subsequent mutations in the ROS1 kinase domain or bypass signaling pathways are frequently the cause of crizotinib resistance. Long-term treatment of ROS1 fusion-positive NSCLC is difficult due to the G2032R solvent front mutation, one of the most prevalent resistance mechanisms [94].

Emerging second-generation TKIs have demonstrated promise in treating crizotinib resistance, including entrectinib and repotrectinib, two next-generation ROS1 inhibitors. A third-generation TKI called lorlatinib has shown promise in treating ROS1-positive NSCLC, including those with brain metastases and those with the G2032R mutation. In patients who had advanced on crizotinib, lorlatinib demonstrated an ORR of 47% in the phase II trial, while individuals with CNS involvement showed long-lasting intracranial responses [95].

Entrectinib, a next-generation TKI, received FDA approval for ROS1-positive NSCLC based on the results from the STARTRK-2 trial, where it demonstrated an ORR of 77% and a median PFS of 19.0 months. Its notable CNS efficacy established it as a standard treatment for ROS1-positive NSCLC, particularly for patients with brain metastases. Entrectinib represented a significant advancement in treating ROS1-positive NSCLC due to its enhanced ability to cross the blood–brain barrier and target CNS metastases, a frequent complication in advanced cases. However, resistance mutations, such as G2032R, have limited their long-term effectiveness, prompting the development of newer inhibitors [96].

Repotrectinib, a next-generation ROS1 inhibitor, has recently become the new standard of care due to its enhanced activity against solvent-front mutations, like G2032R, which render earlier inhibitors ineffective. Repotrectinib has a high activity against ROS1, TRK, and ALK fusions, and has also shown efficacy in both TKI-naïve and TKI-resistant patients, demonstrating significant effectiveness in those with both systemic and CNS disease. In the TRIDENT-1 trial, repotrectinib demonstrated an ORR of 91% in TKI-naïve patients and 44% in those with prior TKI exposure. The drug’s ability to target solvent-front and gatekeeper mutations, which confer resistance to first-line TKIs, makes it a promising option in the treatment-refractory setting. Its strong intracranial activity makes repotrectinib a valuable option for patients with CNS involvement, and its efficacy against ROS1 mutations, like G2032R, gives it a crucial advantage over previous therapies [97].

The development of resistance to ROS1-targeted therapies remains a significant hurdle. In addition to the G2032R mutation, other resistance mechanisms, such as MET amplification and EGFR mutations, have been reported. Ongoing research is focused on understanding these mechanisms and developing novel therapies to overcome resistance [98]. Additionally, ongoing research is focused on developing next-generation ROS1 inhibitors and combination strategies to delay or overcome resistance targeting multiple signaling pathways, such as ROS1 and EGFR or MET, which are being investigated as a strategy to delay or prevent resistance. Immunotherapy in combination with targeted therapies is another area of active research, although its role in ROS1-positive NSCLC remains to be fully elucidated. Novel agents targeting solvent-front mutations, such as taletrectinib, are currently being evaluated in clinical trials [99]. Additionally, combining TKIs with immune checkpoint inhibitors (ICIs) or MET inhibitors represents a potential strategy to circumvent resistance and improve outcomes [100].

In the TRUST-II study, taletrectinib, a potential ROS1 TKI, demonstrated remarkable effectiveness and safety, especially in patients with CNS metastases. Both groups showed robust activity in the research, with TKI-naive patients exhibiting an ORR of 85.2% and TKI-pretreated patients displaying an ORR of 61.7%. With intracranial ORRs of 56.3% in patients who had previously received TKI treatment and 66.7% in patients who had not, taletrectinib has shown notable intracranial activity [101]. Its potential as a top treatment for ROS1-positive NSCLC is highlighted by its good safety profile, which includes a low incidence of serious adverse events [99]. Taletrectinib’s safety and effectiveness in advanced ROS1-positive non-small cell lung cancer were evaluated by the combined analysis of the TRUST-I and TRUST-II trials. Out of the 273 patients assessed, 113 had previously received TKI treatment, and 160 were TKI naïve. Taletrectinib showed a remarkable ORR of 88.8% in the TKI-naïve group, with a median PFS of 45.6 months and a DOR of 44.2 months. The ORR was 55.8%, the median PFS was 9.7 months, and the DOR was 16.6 months for patients who previously had TKI treatment. Significant intracranial efficacy was also demonstrated by the medication; response rates in patients with brain metastases were 56.4% in TKI-pretreated groups and 86.5% in TKI-naïve groups.

Taletrectinib offers durable responses and strong activity, particularly for TKI-naïve patients with ROS1-positive NSCLC, and effectively treats central nervous system involvement. However, resistance mutations, like G2032R, remain a challenge in TKI-pretreated patients, though taletrectinib did show some activity against these mutations. NUV-520, a new ROS1 inhibitor, is under investigation for its potential to address resistant ROS1 mutations, particularly G2032R. Early studies suggest that NUV-520 may offer potent systemic and CNS activity, like repotrectinib and taletrectinib, with a promising safety profile. Although clinical data on NUV-520 are still limited, it holds potential as another option for overcoming resistance mutations and providing durable responses in ROS1-positive patients [102].

ROS1 fusions represent a distinct molecular subset of NSCLC with significant therapeutic implications. The advent of TKIs, such as crizotinib, entrectinib, and lorlatinib, has transformed the management of ROS1 fusion-positive NSCLC, leading to improved survival outcomes. However, the emergence of resistance remains a major challenge. Ongoing research into novel therapies and combination strategies holds promise for overcoming resistance and further improving outcomes for patients with ROS1 fusion-positive NSCLC.

## 7. Conclusions/Perspectives

The discovery of oncogenic fusions, including ALK, NTRK, NRG1, RET, and ROS1 rearrangements, has greatly advanced the therapy of non-small cell lung cancer. Compared to conventional chemotherapy, targeted inhibitors against these fusions have produced more effective treatments with increased efficacy and decreased toxicity. PFS, ORR, and CNS penetration have all been markedly enhanced by second- and third-generation ALK TKIs in comparison to first generation. Even though fusion detection in NSCLC is uncommon, second-generation TRK inhibitors, like selitrectinib and repotrectinib, provide better results with longer-lasting responses to overcome acquired resistance.

NRG1 fusion is a rare type of mutation; however, it represents a clinically significant subset of NSCLC. Despite poor responses to conventional therapies, inhibitors such as afatinib, seribantumab, and zenocutuzumab presented promising responses. The discovery of RET fusions has significant implications for the treatment landscape of oncology, including NSCLC and thyroid cancers. RET inhibitors, such as selpercatinib and pralsetinib, have revealed remarkable efficacy, representing a standard of care therapies. Additionally, developing next-generation TKIs, such as vepafestinib, has shown promise in overcoming resistance mutations. Similarly, next-generation ROS1 inhibitors, such as repotrectinib, and taletrectinib, and emerging agents, like NUV-520, offer promising strategies to overcome resistance mechanisms, particularly solvent-front and gatekeeper mutations.

Despite ongoing newer trials and the emergence of new-generation fusion inhibitors, there are things such as different resistance mechanisms, side effects, and side effects that need to be considered to have better therapeutic effects with a greater quality of life for patients. Future research should focus on overcoming resistance through next-generation inhibitors, combination therapies, and innovative treatment strategies, including immunotherapy and synthetic lethality approaches. Advancements in molecular diagnostics, including liquid biopsy and comprehensive genomic profiling, will further enhance early detection and real-time monitoring of oncogenic fusions. As research continues to refine our understanding of fusion-driven NSCLC, integrating novel targeted therapies with existing treatment modalities will be key to improving patient outcomes and shaping the future of precision medicine in lung cancer.

## Data Availability

No new data were created or analyzed in this study. Data sharing is not applicable to this article.
